# Supplementary material for: Female Sexual Violence: A 12-Year Experience at a Single University Hospital in North-East Italy
Source: Int J Environ Res Public Health. 2024 Mar 5;21(3):301. doi: 10.3390/ijerph21030301 (PMC10969931; doi:10.3390/ijerph21030301)
Supplement: Supplementary file 1 [file ijerph-21-00301-s001.zip › ijerph-2889445-supplementary.pdf]

Supplementary Table S1. Characteristics of sexual abuse and the reported gynecological or physical injuries of all cases, generally.

**General population**	
**Characteristics of sexual violence**	
Range of time to medical help request (days)	6.10 (± 13.97)
SV, one aggressor	145/155 (93.5%)
Group SV	10/155 (6.4%)
Physical violence (added to SV)	20/155 (12.9%)
Disclosure of not penetrative SV	20/155 (12.9%)
Penetration could not be disclosed	38/155 (24.5%)
Victim threatened with a cold weapon	9/155 (5.8%)
Kidnapping	10/155 (6.4%)
Victim's alcohol/drugs abuse before suspected SV	38/155 (24.5%)
Number of toxicological screening tests performed	39/155 (25.1%)
Number of positive toxicological screening tests	23/39 (58.9%)
Number of negative toxicological screening tests	16/39 (41.0%)
Number of positive toxicological screening tests among those who do not remember SV (37 people, 17 tests performed)	12/17 (70.5%)
Suspected use of drug rape	5/155 (3.2%)
Known aggressor	102/155 (65.8%)
Friend/Acquaintance as aggressor	47/102 (46.0%)
Current partner as aggressor	18/102 (17.6%)
Ex partner as aggressor	8/102 (7.8%)
Family member as aggressor	19/102 (18.6%)
Other (such as employer, neighbor)	9/102 (8.8%)
Missing information about the aggressor	1/102 (0.9%)
Unknown aggressor	53/155 (34.1%)
**Clinical evaluation**	
Genital injuries	26/155 (16.7%)
Extragenital injuries	64/155 (41.2%)
No injuries detected	69/155 (44.5%)
Number of STDs screening test performed	138/155 (89.0%)
Number of positive STDs screening tests	63/138 (45.6%)
Number of negative STDs screening tests	75/138 (54.3%)
%HPV infection	49/63 (77.7%)
%HCV	3/63 (4.7%)
%HSV1-2	6/63 (9.5%)
%Chlamydia	7/63 (11.1%)
Number of positive STDs screening tests among those with genital injuries (23 test performed; < 18 yrs: 8 people; > 18 yrs: 15 people)	8/23 (34.7%)
Number of spermatozoa research tests performed	101/155 (65.1%)
Number of positive spermatozoa research tests	28/101 (27.7%)
Number of negative spermatozoa research tests	72/101 (71.2%)
Number of positive spermatozoa research tests among those with genital injuries (17 test performed; < 18 yrs: 5 people; > 18 yrs: 12 people)	3/17 (17.6%)
STDs Prophylaxis	88/155 (56.7%)
Emergency contraceptive administration	53/155 (34.1%)
Refuse of any medical therapy	8/155 (5.1%)
**Victim follow up**	

Psychological-Psichiatric consultation	42/155 (27.0%)
Social worker follow up	16/155 (10.3%)
**Victim discharge**	
Discharge to home	78/155 (50.3%)
Discharge to ER (general ER, pediatric ER)	42/155 (27.0%)
Discharge to another hospital's ward (such as psychiatry, pediatrics)	13/155 (8.3%)
Discharge to anti-violence centre	5/155 (3.2%)
Discharge to police	2/155 (1.2%)
Other (spontaneous departure, return to the community)	6/155 (3.8%)
Missing information	9/155 (5.8%)
Mean duration of medical service (hours)	2.84

Legend:
SV: sexual violence
STDs: sexually transmitted diseases
HPV: Human Papillomavirus
HCV: Hepatitis C virus
HSV1-2: Herpes Simplex virus 1-2
ER: emergency room
